# Parainfectious encephalomyeloradiculitis associated with bacterial meningitis: a case report

**DOI:** 10.1186/s13256-014-0508-1

**Published:** 2015-02-10

**Authors:** Yuji Kato, Takeshi Hayashi, Akira Uchino, Ichiro Deguchi, Norio Tanahashi

**Affiliations:** 1 Department of Neurology and Cerebrovascular Medicine, Saitama International Medical Center, Saitama Medical University, 1397-1 Yamane, Hidaka, Saitama 350-1298 Japan; 2 Department of Diagnostic Radiology, Saitama International Medical Center, Saitama Medical University, 1397-1 Yamane, Hidaka, Saitama 350-1298 Japan

**Keywords:** Acute disseminated encephalomyelitis, Bacterial meningitis, Encephalomyeloradiculitis, Polyradiculoneuropathy, Post-infectious

## Abstract

**Introduction:**

Acute disseminated encephalomyelitis in adulthood occurs in most cases after a viral infection. Acute disseminated encephalomyelitis associated with bacterial meningitis, however, is quite rare.

**Case presentation:**

An 82-year-old Japanese woman presented with a fever and somnolence. Increased neutrophil count and protein content, and decreased glucose levels in her cerebrospinal fluid initially suggested bacterial meningitis. Brain magnetic resonance imaging on admission showed bilateral symmetrical lesions in her brainstem and her cerebellum. She was diagnosed with acute disseminated encephalomyelitis following bacterial meningitis. Even though appropriate antibiotic and steroid treatment improved her symptoms, she developed transverse myelitis and lumbosacral polyradiculitis on day 9.

**Conclusions:**

Parainfectious encephalomyeloradiculitis, a variant of acute disseminated encephalomyelitis, is a unique neurological syndrome that may be caused by bacterial infection in the central nervous system.

## Introduction

Acute disseminated encephalomyelitis (ADEM) in adulthood occurs in most cases after a viral infection [1]. *Mycoplasma pneumoniae* infection or vaccinations can precede it in some cases [1]. ADEM associated with bacterial meningitis, however, is quite rare. The occurrence of ADEM with peripheral nervous system (PNS) involvement, mainly with features of polyradiculoneuropathy, is currently considered to be more frequent than it was previously [2]. We present a case of parainfectious encephalomyeloradiculitis (EMR), a variant of ADEM, following bacterial meningitis.

## Case presentation

**Figure 1:**
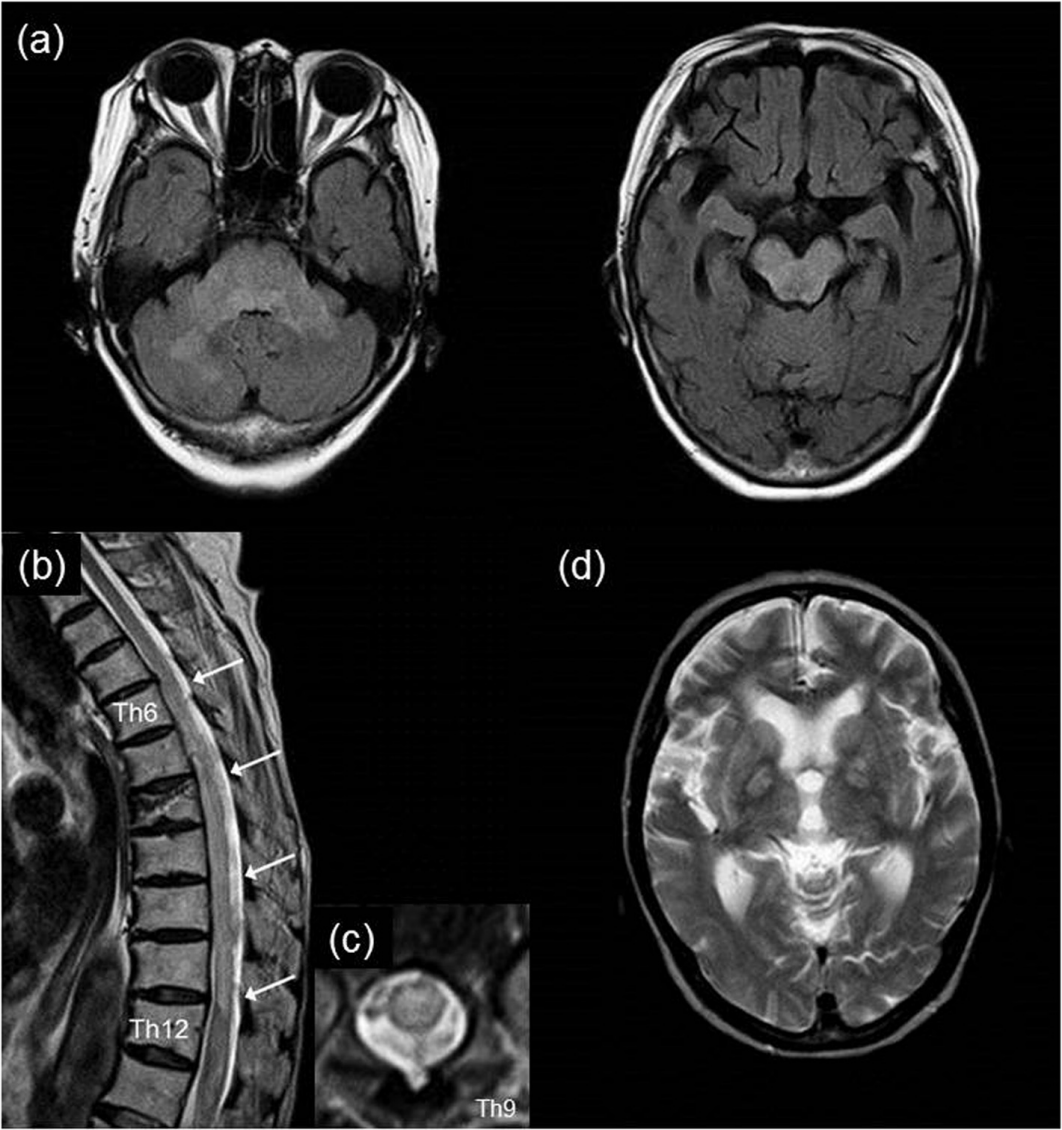
**Brain magnetic resonance imaging performed on admission.** Axial fluid-attenuated inversion recovery images showed bilateral symmetric hyperintensity in the brainstem and cerebellum **(a)**. Magnetic resonance imaging of the spinal cord performed on day 11. Sagittal T2-weighted image revealed an intramedullary lesion from the level of the 6th to the 12th thoracic vertebrae (**b**, arrows). There is compression fracture in the 8th thoracic vertebra. Axial T2-weighted image at 9th thoracic vertebrae indicated transverse myelitis **(c)**. T2-weighted magnetic resonance imaging on day 19 showed bilateral symmetric high-intensity lesions in the globus pallidus **(d)**. Abbreviation: Th, thoracic.

An 82-year-old Japanese woman experienced chills, a wet cough, and vertigo for 3 days. She had received intravenous ceftriaxone in another institution. She developed fever and disturbed consciousness and was then admitted to our institution. Her past medical history included chronic obstructive pulmonary disease. There was no history of recent vaccination. A physical examination revealed a high fever and labored breathing. On neurological examination, she was somnolent and had neck stiffness. Her oculocephalic responses were normal, her muscle power, tone, and tendon reflexes were normal, and Babinski reflex was absent. Laboratory investigations on admission revealed the following: a white blood cell count of 13,300/μL (88.7% neutrophils) and C-reactive protein of 16.0mg/dL. A lumbar puncture yielded xanthochromic cerebrospinal fluid (CSF) containing 615 cells/μL (95% polymorphonuclear cells, 5% mononuclear cells), an elevated protein content of 243mg/dL, a low glucose level of 37mg/dL (serum glucose level 122mg/dL), and increased myelin basic protein of 511pg/mL. Culture of her blood and CSF produced no bacteria. Cytology of her CSF was class II, which meant reactive pleocytosis with no evidence of malignancy. Brain magnetic resonance imaging (MRI) showed bilateral symmetrical lesions in her brainstem and her cerebellum (Figure 1a). Magnetic resonance angiography revealed no abnormalities. Thoracoabdominal computed tomography revealed no infectious lesions. ADEM following bacterial meningitis was suspected; intravenous meropenem and dexamethasone were immediately initiated as an empirical treatment for bacterial meningitis.

The patient improved over the next few days. However, on day 9, she suddenly developed flaccid paralysis of her lower limbs, associated with sensory loss below the level of her ninth thoracic vertebra and urinary retention. Tendon reflexes were normal in her upper extremities, but absent in her lower extremities. MRI of her spinal cord performed on day 11 revealed an intramedullary lesion at the level of the 6th to 12th thoracic vertebrae (Figure 1b, c), and high-dose corticosteroid therapy was started. After 18 days of antibiotic therapy, a repeat lumbar puncture showed an improvement in her CSF findings: 11 cells/μL, a protein content of 10mg/dL, and glucose levels of 98mg/dL (serum glucose 121mg/dL). By contrast, her symptoms did not improve. A brain MRI on day 19 showed resolution of the prior lesions and the development of new lesions in her basal ganglia (Figure 1d). On day 23, a nerve conduction study was performed. This showed that motor nerve conduction velocity (37.0m/second) and compound muscle action potential (3.2mV) in her tibial nerve were decreased, and an F wave could not be evoked. Those in her median nerve were normal. These results suggested parainfectious EMR. After a month, her flaccid paraplegia was only slightly alleviated.

## Discussion

In the present case, the clinical course and laboratory data were consistent with partially treated bacterial meningitis, although the causative bacterium was not identified. Some bacterial agents are associated with ADEM, although less frequently than viruses. These bacteria include *Streptococcus* [3], *Mycoplasma pneumoniae* [1] and *Haemophilus influenzae* [4]. Acute EMR is a unique neurological syndrome that was reported by Marrie *et al*. [5] and Kusuhara *et al*. [6].

Acute EMR was defined by febrile illness followed, within days, by neurological illness that consisted of neurogenic bladder, paraparesis, and altered consciousness. CSF reveals pleocytosis and nerve conduction study reveals polyradiculopathy [5,6]. The other clinical features were brain stem or cranial nerve involvement [5,6]. In ADEM, involvement of the PNS has previously been described as a rare variant, but Marchioni *et al*. reported that 43.6% of patients with ADEM showed PNS involvement in the form of polyradiculoneuropathy [2]. In their studies, old age, female gender, spinal cord involvement, and PNS damage predicted a worse outcome [2]. Our patient exhibited these features, which was consistent with the poor recovery.

Prior study has shown that streptococcal exotoxins can induce T-cell mediated antimyelin autoreactivity, consistent with the demyelinating lesions seen in ADEM [7]. Although speculative, it is a possible explanation of the pathogenesis in this case.

For the first time to the best of our knowledge, we have shown here that EMR can occur in association with bacterial meningitis. Profound CSF leukocytosis suggests implication of bacterial infection, because ADEM preceded by viral infection usually shows only mild to moderate pleocytosis [1,8]. Prompt diagnosis and treatment would improve the prognosis of EMR after bacterial infection.

## Conclusion

Parainfectious EMR, a variant of ADEM, is a unique neurological syndrome that may be caused by bacterial infection in the central nervous system.

## Consent

Written informed consent was obtained from the patient for publication of this case report and accompanying images. A copy of the written consent is available for review by the Editor-in-Chief of this journal.

## Abbreviations

ADEM: Acute disseminated encephalomyelitis
CSF: Cerebrospinal fluid
EMR: Encephalomyeloradiculitis
MRI: Magnetic resonance imaging
PNS: Peripheral nervous system
